# The Role of CyberKnife Stereotactic Radiosurgery in Recurrent Cranial Medulloblastomas across Pediatric and Adult Populations †

**DOI:** 10.3390/jcm13123592

**Published:** 2024-06-19

**Authors:** Kelly H. Yoo, Neelan J. Marianayagam, David J. Park, Aroosa Zamarud, Xuejun Gu, Erqi Pollom, Scott G. Soltys, Antonio Meola, Steven D. Chang

**Affiliations:** 1 Department of Neurosurgery, Stanford University School of Medicine, Stanford, CA 94304, USA; njm39@stanford.edu (N.J.M.); djpark@stanford.edu (D.J.P.); azamarud@stanford.edu (A.Z.); a.meola@hotmail.it (A.M.); 2 Department of Radiation Oncology, Stanford University School of Medicine, Stanford, CA 94304, USA; xuejungu@stanford.edu (X.G.); epollom@stanford.edu (E.P.); sgsoltys@stanford.edu (S.G.S.)

**Keywords:** CyberKnife radiosurgery, medulloblastoma, stereotactic radiosurgery

## Abstract

**Background/Objectives**: Medulloblastoma is the most common malignant brain tumor in children. In recent decades, the therapeutic landscape has undergone significant changes, with stereotactic radiosurgery (SRS) emerging as a promising treatment for recurrent cases. Our study provides a comprehensive analysis of the long-term efficacy and safety of SRS in recurrent medulloblastomas across both pediatric and adult patients at a single institution. **Methods**: We retrospectively reviewed the clinical and radiological records of patients who underwent CyberKnife SRS for recurrent cranial medulloblastomas at our institution between 1998 and 2023. Follow-up data were available for 15 medulloblastomas in 10 patients. The cohort comprised eight pediatric patients (ages 3–18) and two adult patients (ages 19–75). The median age at the time of SRS was 13 years, the median tumor volume accounted for 1.9 cc, the median biologically equivalent dose (BED) was 126 Gy, and the single-fraction equivalent dose (SFED) was 18 Gy. The SRS was administered at 75% of the median isodose line. **Results**: Following a median follow-up of 39 months (range: 6–78), 53.3% of the medulloblastomas progressed, 13.3% regressed, and 33.3% remained stable. The 3-year local tumor control (LTC) rate for all medulloblastomas was 65%, with lower rates observed in the adult cohort (50%) and higher rates in pediatric patients (67%). The 3-year overall survival (OS) rate was 70%, with significantly higher rates in pediatric patients (75%) compared to adult patients (50%). The 3-year progression-free survival (PFS) rate was 58.3%, with higher rates in pediatric patients (60%) compared to adult patients (50%). Two pediatric patients developed radiation-induced edema, while two adult patients experienced radiation necrosis at the latest follow-up, with both adult patients passing away. **Conclusions**: Our study provides a complex perspective on the efficacy and safety of CyberKnife SRS in treating recurrent cranial medulloblastomas across pediatric and adult populations. The rarity of adverse radiation events (AREs) underscores the safety profile of SRS, reinforcing its role in enhancing treatment outcomes. The intricacies of symptomatic outcomes, intertwined with factors such as age, tumor location, and prior surgeries, emphasize the need for personalized treatment approaches. Our findings underscore the imperative for ongoing research and the development of more refined treatment strategies for recurrent medulloblastomas. Given the observed disparities in treatment outcomes, a more meticulous tailoring of treatment approaches becomes crucial.

## 1. Introduction

First described in 1925, medulloblastoma remains a formidable malignant brain neoplasm primarily affecting the pediatric population, with a peak age of diagnosis typically between 6 and 8 years [1]. While the conventional therapeutic triad of surgery, chemotherapy (CT), and radiation therapy (RT) has historically formed the cornerstone of management [2,3], recent decades have witnessed significant paradigm shifts in therapeutic strategies. These shifts have been propelled by an enhanced comprehension of molecular subgroups influencing both prognosis and treatment modalities [4]. Nevertheless, the management of recurrent cases continues to present formidable clinical challenges, underscoring the aggressive nature of this disease and the imperative for effective interventions [5].

The intricate interplay between the infiltrative nature of medulloblastoma and the intricacies of neural architecture necessitates precise and efficacious therapeutic strategies [6]. Clinical challenges associated with recurrent medulloblastoma, such as treatment resistance, neurocognitive impairments, and compromised quality of life for affected individuals and their families, highlight the urgent need for therapeutic approaches aimed at enhancing patient outcomes and mitigating long-term sequelae [5].

In this context, stereotactic radiosurgery (SRS) has emerged as a promising treatment modality for managing recurrent medulloblastoma [7]. By leveraging targeted ionizing radiation, SRS strategically exploits the biological vulnerabilities of the pathology, offering the advantage of delivering precisely targeted radiation to tumor sites while sparing surrounding healthy tissue, thereby minimizing treatment-related toxicities [8]. Supported by advancements in radiological imaging, radiation delivery platforms, and an evolving understanding of radiobiology, SRS has assumed a pivotal role in combating this challenging adversary [9]. The rationale for studying SRS in the context of recurrent medulloblastoma is rooted in its potential to address the limitations of conventional treatment approaches and provide durable control of disease progression.

Our study aims to investigate the long-term efficacy and safety of SRS for both pediatric and adult patients with recurrent medulloblastoma. Situated within a single institution, our investigation integrates clinical expertise with research acumen, furnishing a comprehensive perspective and evaluation of the multifaceted dimensions of SRS application and outcomes in this challenging patient population. By elucidating the clinical impact of SRS and identifying factors predictive of treatment response, our findings have the potential to inform treatment decision-making, contribute to the refinement of current management guidelines, and pave the way for future advancements in the management of recurrent medulloblastoma.

## 2. Methods

### 2.1. Patient Selection and Characteristics

Between 1998 and 2023, we identified and treated 15 cases of recurrent medulloblastomas in 10 patients at Stanford University Medical Center using CyberKnife SRS. Inclusion criteria comprised patients with recurrent medulloblastomas who underwent CyberKnife SRS and had a minimum follow-up period of 6 months. Clinical data were collected and securely stored in a database approved by the Institutional Review Board (IRB) #3019 at Stanford University, adhering to principles outlined in the Helsinki Declaration. All participants provided informed consent for the use of their data in research aimed at improving future patient care. Access to patient data is restricted to authorized personnel only. Data are available upon request from the first author, KHY, and are not publicly accessible to ensure compliance with Health Insurance Portability and Accountability Act (HIPAA) regulations and safeguard patient privacy.

The study cohort comprised eight (80%) pediatric patients (ages 3–18) and two (20%) adult patients (ages 19–75). The median age at the time of SRS was 13 years, with the pediatric group having a median age of 12 years (range: 9–16) and the adult group with a median age of 27 years (range: 25–29). Nine (90%) patients underwent surgical resection prior to SRS, with histological confirmation of WHO grade IV tumors. Additionally, one (10%) adult patient received RT as primary treatment based on radiographic diagnosis. Symptoms observed in patients were correlated with lesion location and included headaches, ataxia, nausea, vomiting, seizures, visual impairment, and motor impairment. During the follow-up period, one patient passed away, while there have been no instances of patients lost to follow-up thus far (Table 1, Supplementary Table S1).

### 2.2. Tumor Characteristics

This study included 10 patients with 15 recurrent cranial medulloblastomas. The median tumor volume was 1.9 cc (mean: 2.7; range: 0.02–8.7). In the pediatric group, the median tumor volume was 1.9 cc (range: 0.02–8.7), while, in the adult group, it was 1.5 cc (range: 0.5–2.5). Most lesions (86.7%) occurred in pediatric patients, primarily localized in the cerebellum (*n* = 5, 33.3%), followed by the ventricular (*n* = 2, 13.3%), parietal (*n* = 2, 13.3%), and frontal (*n* = 2, 13.3%) regions (Table 1).

Considering the protracted timeline of our study cohort, encompassing the period from 1998 to 2023, acquiring comprehensive molecular subgroup data, specifically pertaining to WNT and SHH subtypes, for all patients, particularly those diagnosed earlier in the timeline, posed significant challenges. Nonetheless, a thorough investigation of the histopathological characteristics of our recurrent medulloblastoma cohort was conducted. Notably, we noted a consistent trend toward features suggestive of anaplastic subtypes or focal anaplasia across our patient population. Furthermore, all cases were consistently categorized as high-risk and assigned a WHO grade IV classification.

Recurrence was defined as the reappearance or progression of the tumor following primary treatment, typically surgical resection. Patients undergoing regular follow-up evaluations after primary treatment were systematically monitored clinically and radiographically. The collected data were rigorously evaluated by interdisciplinary professional colleagues to either confirm or exclude the diagnosis of recurrent medulloblastoma.

### 2.3. Treatment

Cranial recurrent medulloblastomas were treated with CyberKnife SRS (Accuray, Inc., Sunnyvale, CA, USA) utilizing the established method previously outlined by Shi et al. [10]. The median time between initial diagnosis and SRS was 30 months (range: 12–108), with pediatric patients undergoing treatment at a median of 39 months (range: 12–108) and adult patients at a median of 24.5 months (range: 24–25). SRS was administered with a median single-fraction equivalent dose (SFED) of 18 Gy to the 75% median isodose line (range: 65–81). Treatment parameters, including marginal dose, maximum dose, isodose line, and the number of fractions, did not significantly differ between cohorts (Table 2).

### 2.4. Biologically Effective Dose (BED), Single-Fraction Equivalent Dose (SFED), and Equivalent Total Doses in 2-Gy Fractions (EQD2)

The biologically effective dose (BED) was calculated using the linear-quadratic (LQ) model with an α/β ratio of 3 Gy, following established methods outlined in previous studies [11,12]. BED values were subsequently converted to SFED and equivalent total doses in 2-Gy fractions (EQD2) using the LQ model [13]. The median BED, SFED, and EQD2 for the entire cohort were calculated as 126 Gy (range: 66.7–153.3), 18 Gy (range: 12.7–20), and 75.6 Gy (range: 40–92), respectively. Importantly, no significant differences were observed in these parameters between patient groups (Table 2).

### 2.5. Follow-Up

Following CyberKnife SRS for recurrent medulloblastomas, patients underwent clinical evaluation and MRI follow-up semi-annually during the first two years post-treatment, followed by annual assessments thereafter. Clinical evaluations included comprehensive neurological examinations to assess symptoms and treatment response.

MRI scans were performed using standardized imaging protocols, and tumor volume was measured in the axial, sagittal, and coronal planes by experienced radiologists. The tumor volume was estimated as (D1 × D2 × D3)/2, where D represents the maximum diameter of the tumor in each plane. This approach ensured consistent and accurate assessment of tumor size over time.

Criteria for tumor response assessment were as follows: an increase of more than 10% of the initial treatment volume or tumor occurrence outside the planned treatment volume was considered tumor progression, while a decrease in volume greater than 10% was defined as tumor regression. Stable disease was characterized by tumor dimensions within 10% of the volume at the time of SRS treatment. Local tumor control was determined based on reduction or stability in tumor size.

Data from follow-up assessments were analyzed using Kaplan–Meier analysis to evaluate patient outcomes. Graphical representations were generated using standard statistical software (IBM SPSS Statistics 29.0, Chicago, IL, USA).

## 3. Results

### 3.1. Patient Demographics and Characteristics

Following the exclusion of patients lost to follow-up, the study encompassed 15 lesions observed in 10 patients. Among these, 80% (*n* = 8) were pediatric and 20% (*n* = 2) were adults. Over the evaluation period, 50% (*n* = 5) of the patients succumbed. The median follow-up duration post-SRS was 39 months (range: 6–78) (Table 2).

### 3.2. Local Tumor Control

**Figure 1:**
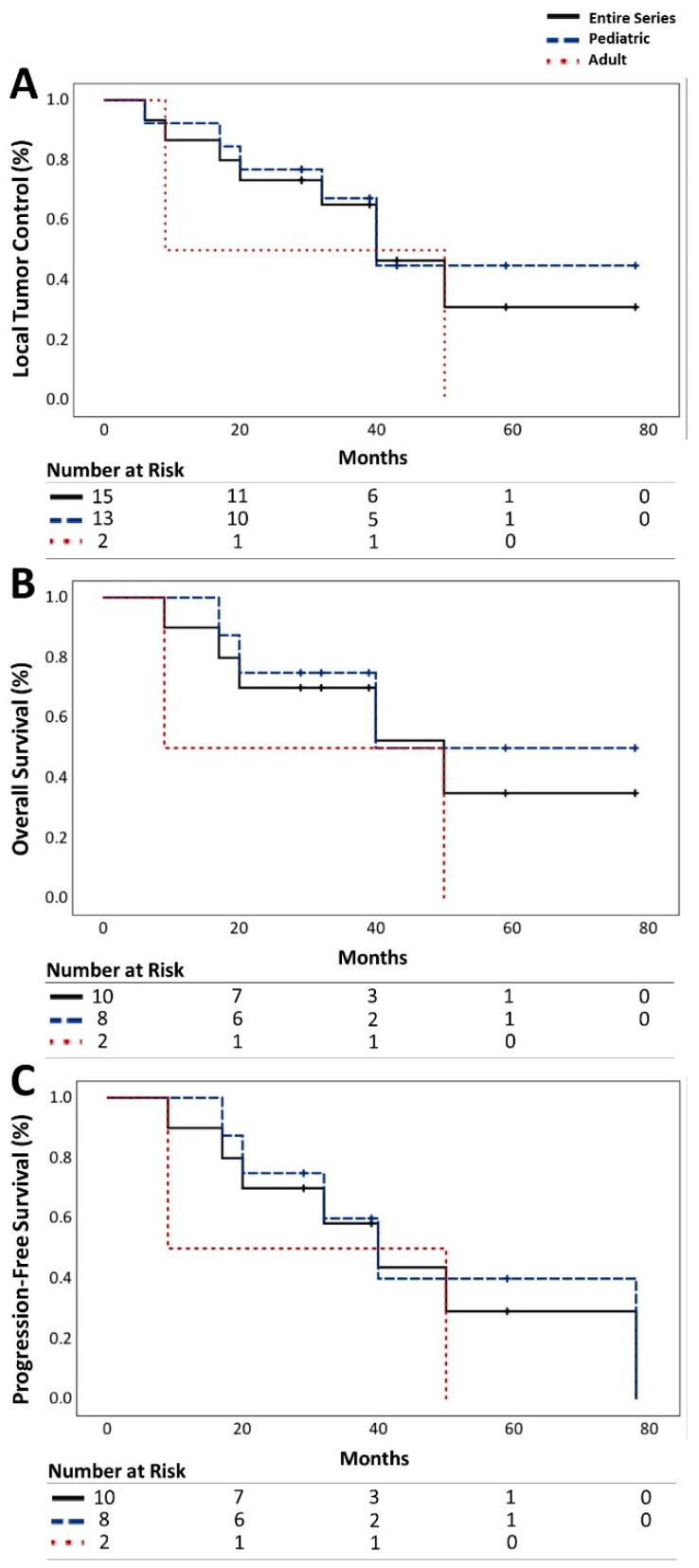
Summary of patient outcome according to Kaplan–Meier method after treatment with CyberKnife SRS. (**A**) Local tumor control (LTC) rate, (**B**) overall survival (OS) rate, and (**C**) progression-free survival (PFS) rate of the adult (black, solid) and pediatric (blue, dash) series with recurrent medulloblastomas with number of lesions (**A**) and patients (**B** + **C**) at risk.

Among the 15 recurrent medulloblastomas, 53.3% (*n* = 8) demonstrated progression, 13.3% (*n* = 2) exhibited radiographic regression, and 33.3% (*n* = 5) remained stable. The LTC rates for all medulloblastomas were 86%, 65%, and 31% at 1, 3, and 5 years, respectively. Notably, pediatric patients showed a higher LTC rate of 67% at 3 years compared to the adult cohort, which stood at 50% (Table 3, Figure 1A).

Further analysis of LTC rates revealed intriguing patterns associated with tumor location. Cerebellar lesions displayed a favorable response with a 3-year LTC rate of 78% compared to ventricular lesions, which exhibited a rate of 50%. Furthermore, LTC rates showed a correlation with the number of prior surgeries, with a higher rate observed in patients who had undergone a single surgery (75%) compared to those with multiple surgeries (66.7%) (Table 1 and Table 3).

### 3.3. Patient Survival

OS rates for all patients stood at 90%, 70%, and 35% at 1, 3, and 5 years, respectively. Pediatric patients exhibited higher rates of OS (75%) compared to adult patients (50%) at the 3-year mark (Table 3, Figure 1B).

Moreover, survival outcomes were influenced by clinical presentation. Asymptomatic patients demonstrated a 100% OS rate at 3 years, whereas symptomatic patients exhibited varied rates based on the location of the tumor (Table 1 and Table 3).

### 3.4. Progression-Free Survival

PFS rates were 90%, 58%, and 29% for all patients at 1, 3, and 5 years, respectively, with a higher rate observed in the pediatric cohort at 3 years (60%) (Table 3, Figure 1C).

Notably, PFS was significantly influenced by the age at treatment, with pediatric patients experiencing a 60% PFS rate at 3 years compared to 50% in adults. Additionally, a positive correlation was observed between PFS and target tumor volume, with larger volumes exhibiting a slightly higher PFS rate (Table 2 and Table 3).

### 3.5. Symptomatic Outcome

Among the entire cohort, one (10%) pediatric patient was asymptomatic, allowing the evaluation of symptomatic outcomes for nine (90%) patients with 14 (93.3%) symptomatic medulloblastomas. Overall, seven (77.8%) patients demonstrated symptomatic improvements following SRS (Table 3).

Significantly, symptomatic improvement was more pronounced in the pediatric cohort (75%) compared to the adult cohort (50%) (*p* = 0.05). These improvements encompassed relief from headaches, nausea, vomiting, ataxia, and visual impairment. However, one (12.5%) pediatric patient and one (50%) adult patient experienced worsening headaches, while one (50%) adult patient presented with new symptoms, including dizziness and vomiting post-SRS (Table 3).

Symptomatic outcomes were notably associated with the location of tumors, with cerebellar lesions showing the highest rate of improvement (80%). Furthermore, improvements in symptoms were more evident in cases with a single prior surgery compared to multiple surgeries (81.3% vs. 57.1%) (Table 1, Table 2 and Table 3).

### 3.6. Adverse Radiation Effect (ARE)

Among the entire cohort, two pediatric patients experienced radiation-induced edema, while two adult patients were diagnosed with radiation necrosis. Patients who experienced AREs exhibited similar dose parameters as the overall cohort, underscoring the importance of exploring individual factors that may increase susceptibility. Factors such as genetic predisposition, underlying medical conditions, and variations in tissue response to radiation may influence the likelihood and severity of AREs. Further research is warranted to elucidate the underlying mechanisms of AREs and identify strategies for risk stratification and prevention.

## 4. Discussion

**Figure 2:**
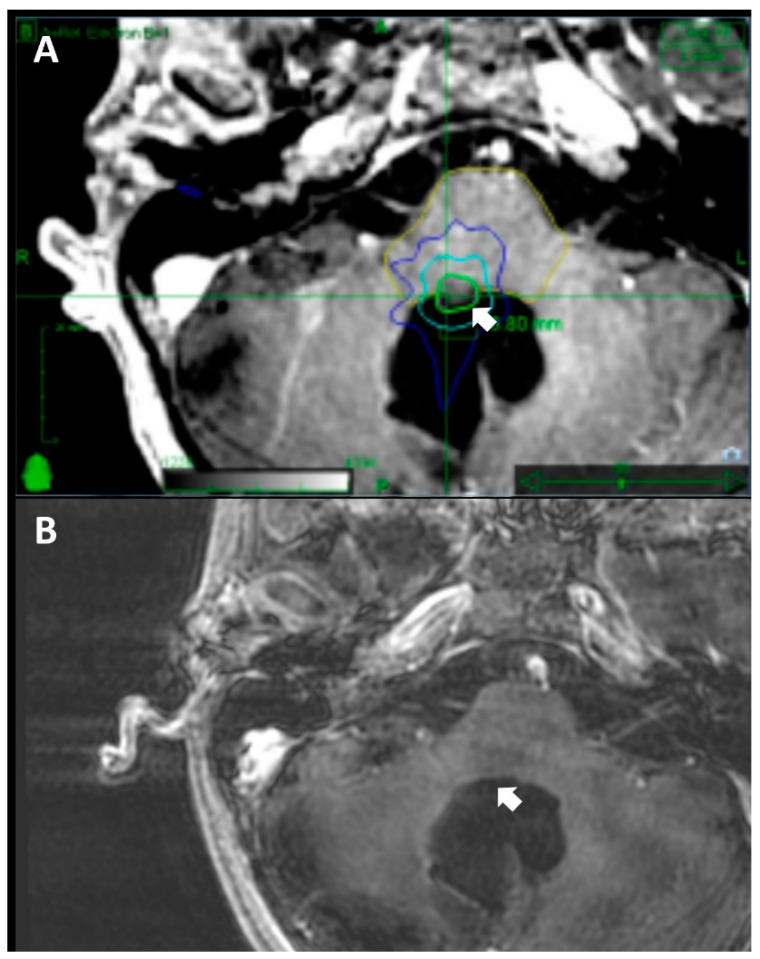
Comparison of (**A**) a baseline CyberKnife treatment plan and (**B**) the most recent radiographic follow-up evaluation of an 11-year-old male patient with a cervicomedullary recurrent medulloblastoma status post-surgical resection and adjuvant radiation therapy. A marginal dose of 18 Gy was delivered with the maximum dose of 24.16 Gy in a single fraction to 75% of the isodose line (**A**) A substantial reduction in tumor size was evident in the 41-month follow-up MRI (**B**). The arrows indicate the location of the tumor at treatment initiation and its disappearance at the latest follow-up, highlighting key information central to this figure and the manuscript.

Our investigation into the outcomes of SRS for recurrent cranial medulloblastomas presents a thorough analysis of treatment efficacy and patient management (Figure 2). The observed variations in treatment outcomes between pediatric and adult patients underscore the necessity for tailored treatment approaches. This divergence may be attributed to factors such as enhanced central nervous system (CNS) plasticity in pediatric patients or the presence of less aggressive tumor types [14]. By integrating demographic, clinical, and treatment factors, our study provides a nuanced understanding of the intricate landscape associated with recurrent medulloblastomas.

### 4.1. Local Tumor Control

The observed LTC rates, especially the substantial 67% rate at 3 years in the pediatric cohort, emphasize the potential efficacy of SRS in managing recurrent medulloblastomas. These findings align with the existing literature, emphasizing the significance of precision in targeting specific tumor locations, particularly cerebellar lesions, to optimize treatment outcomes [7,15,16,17,18].

Pediatric patients, constituting a majority in our cohort, demonstrate a higher prevalence of cerebellar lesions, which may contribute to the enhanced LTC rates observed in this subgroup (Table 1). Conversely, the higher prevalence of multiple prior surgeries in the adult cohort may be associated with comparatively lower LTC rates, suggesting a potential link between prior surgical intervention and LTC outcomes [19,20,21]. Further exploration of associations between the number of fractions, BED, and LTC rates, considering factors such as age and tumor location, could enhance our understanding of the interplay influencing LTC outcomes.

### 4.2. Patient Survival and Progression-Free Survival

The OS rates of 90%, 70%, and 35% at 1, 3, and 5 years underscore the potential long-term benefits of SRS in managing recurrent cranial medulloblastomas, especially given their aggressive nature [22]. The notably higher survival rates in pediatric patients compared to their adult counterparts emphasize age as a pertinent prognostic factor [23,24]. Moreover, the higher prevalence of multiple prior surgeries in the adult cohort may contribute to the lower OS rates, emphasizing the impact of prior treatment history on long-term survival outcomes [25]. Shorter intervals between diagnosis and SRS may be associated with more favorable outcomes, suggesting that prompt initiation of SRS after diagnosis could contribute to improved survival rates. Smaller tumor volumes may correlate with greater OS and PFS outcomes, highlighting the importance of accurately defining and targeting tumor boundaries during SRS [26]. Optimal margin doses play a crucial role in improving OS and PFS, with deviations potentially impacting outcomes [27].

The nuanced interplay of these factors underscores the need for personalized treatment strategies, tailoring therapeutic approaches based on individual patient characteristics to optimize treatment outcomes.

### 4.3. Symptomatic Outcomes

Our investigation of symptomatic outcomes post-SRS reveals notable improvements, particularly in the pediatric patient cohort. With a higher percentage experiencing SI and lower rates of SW and NS, a potential correlation between clinical response and long-term survival outcomes is suggested [28].

Pediatric patients, constituting 80% of our cohort, demonstrate a particularly encouraging trend in symptomatic outcomes [29,30]. The SI percentage of 75% in the pediatric group, compared to 50% in adults, underscores the positive impact of SRS on symptom relief in younger patients [31]. Moreover, the correlation between tumor location and SI emphasizes the need for tailored treatment approaches, with targeting cerebellar lesions yielding favorable symptomatic outcomes.

The influence of the number of prior surgeries on symptomatic outcomes necessitates a critical examination of the optimal timing and extent of surgical interventions in managing recurrent medulloblastomas [30,32]. While SRS contributes to SI, understanding the interplay between prior surgeries and post-SRS symptoms is crucial for refining treatment strategies [33]. Our findings suggest that a judicious approach to surgical interventions, considering factors such as timing and extent, may contribute to more favorable symptomatic outcomes in the context of recurrent medulloblastomas.

### 4.4. Adverse Radiation Effects

The identification of radiation-induced edema and necrosis, albeit in a subset of patients, emphasizes the critical role of meticulous dose planning [29,34]. These occurrences in both pediatric and adult patients underscore the imperative for personalized treatment strategies to mitigate ARE [1].

Furthermore, the absence of myelopathy provides reassurance and aligns with the safety profile reported in the existing literature [35,36,37,38].

### 4.5. Integration with Previous Studies

Our findings align closely with prior studies investigating the role of SRS in recurrent medulloblastomas, reinforcing the essential role of a multidisciplinary approach in patient care [4,31]. The alignment with the established literature enhances the generalizability of our results and significantly contributes to the growing body of evidence supporting the efficacy of SRS in managing recurrent cranial medulloblastomas.

### 4.6. Limitations and Future Directions

While our study offers promising insights into the efficacy and safety of CyberKnife SRS for recurrent medulloblastomas, several limitations merit consideration. The retrospective design and the relatively low sample size underscore the need for a cautious interpretation of our findings. Prospective studies with larger cohorts are imperative to validate our results and strengthen the evidence base.

Moreover, despite the comprehensive nature of our follow-up protocol, the relatively short duration of follow-up in some cases limits our ability to assess the long-term durability of treatment responses and potential late effects. Sustained long-term follow-up may be pivotal for elucidating the long-term efficacy and safety profile of CyberKnife SRS in this patient population.

In addition to these limitations, several areas warrant further investigation in future research endeavors. Firstly, comparative studies that evaluate the efficacy and safety of CyberKnife SRS against other treatment modalities commonly used for recurrent medulloblastomas, such as conventional radiotherapy and surgical resection, would provide valuable insights into optimal treatment strategies. Secondly, future research should explore novel treatment strategies, identify biomarkers for predicting treatment response in terms of combination therapy, and investigate personalized medicine approaches in the management of recurrent medulloblastomas. These avenues hold the potential to further improve patient outcomes and advance our understanding of this challenging disease.

By acknowledging these limitations and delineating future research directions, we aim to lay the groundwork for ongoing investigations that build upon our initial insights and ultimately enhance the care of patients with recurrent medulloblastomas.

## 5. Conclusions

Our study provides a nuanced perspective on the efficacy and safety of CyberKnife SRS in addressing recurrent cranial medulloblastomas across pediatric and adult demographics. The low incidence of ARE accentuates the favorable safety profile associated with CyberKnife SRS, reaffirming its role in optimizing treatment outcomes. The intricacies of symptomatic responses, influenced by variables such as age, tumor localization, and prior surgical interventions, underscore the imperative for tailored therapeutic strategies. Our results underscore the ongoing research endeavors and the development of more refined and effective treatment approaches for recurrent medulloblastomas. Given the observed disparities in treatment outcomes between pediatric and adult cohorts, meticulous customization of treatment modalities becomes crucial. 

## Figures and Tables

**Table 1:** Demographic characteristics in 10 patients with 15 recurrent cranial medulloblastomas.

Characteristics	Entire Cohort	Pediatric	Adult
**# patients**	**10 (100%)**	**8 (80%)**	**2 (20%)**
* Sex*			
Male	8 (80%)	6 (75%)	2 (100%)
Female	2 (20%)	2 (25%)	0 (0%)
* Prior Surgery*			
Single	6 (60%)	6 (75%)	0 (0%)
Multiple	3 (30%)	1 (12.5%)	2 (100%)
Symptoms			
Headaches	6 (60%)	4 (50%)	2 (100%)
Nausea	4 (40%)	4 (50%)	1 (50%)
Vomiting	5 (50%)	4 (50%)	1 (50%)
Ataxia	3 (30%)	1 (12.5%)	2 (100%)
Visual impairment	3 (30%)	2 (25%)	1 (50%)
Seizure	2 (20%)	2 (25%)	0 (0%)
Left-sided dysmetria	1 (10%)	1 (12.5%)	0 (0%)
Peripheral rigidity	1 (10%)	1 (12.5%)	0 (0%)
**# tumors**	**15 (100%)**	**13 (86.7%)**	**2 (13.3%)**
* Location*			
Cerebellar	5 (33.3%)	4 (30.8%)	1 (50%)
Ventricular	2 (13.3%)	1 (7.7%)	1 (50%)
Frontal	2 (13.3%)	2 (15.4%)	0 (0%)
Parietal	2 (13.3%)	2 (15.4%)	0 (0%)
Temporal	1 (6.7%)	1 (7.7%)	0 (0%)
Thalamic	1 (6.7%)	1 (7.7%)	0 (0%)
Cervicomedullary	1 (6.7%)	1 (7.7%)	0 (0%)
Medullary	1 (6.7%)	1 (7.7%)	0 (0%)
* Clinical Presentation*			
Symptomatic	14 (93.3%)	12 (92.3%)	2 (100%)
Asymptomatic	1 (6.7%)	1 (7.7%)	0 (0%)

*#, numbers.*

**Table 2:** Treatment characteristics in 10 patients with 15 recurrent cranial medulloblastomas.

Characteristics	Entire Cohort	Pediatric	Adult	Statistical Significance (*p* Values)
**# Tumors per patient**				**0.4**
Mean	1.5	1.6	1	
Median	1	1	1	
Range	1–4	1–4	1	
**Age at Treatment, yrs**				
Mean	14.3	12.4	27	**<0.001**
Median	13	12	27	
Range	9–29	9–16	25–29	
**Interval between Diagnosis to SRS, mo**				
Mean	42.7	45.5	24.5	
Median	30	39	24.5	**0.4**
Range	12–108	12–108	24–25	
**Target Tumor Volume, cc**				
Mean	2.7	2.9	1.5	0.51
Median	1.9	1.8	1.5	
Range	0.02–8.7	0.02–8.7	0.5–2.5	
**Margin Dose, Gy**				
*1 Fraction*				0.8
Mean	17.8	17.8	18	
Median	18	18	18	
Range	14–20	14–20	18	
*2 Fractions*				
Mean	20	20	N/A	
Median	20	20	N/A	
Range	20	20	N/A	
*3 Fractions*				
Mean	N/A	N/A	N/A	
Median	N/A	N/A	N/A	
Range	N/A	N/A	N/A	
*4 Fractions*				
Mean	N/A	N/A	N/A	
Median	N/A	N/A	N/A	
Range	N/A	N/A	N/A	
*5 Fractions*				
Mean	25	25	N/A	
Median	25	25	N/A	
Range	25	25	N/A	
**Maximum Dose, Gy**				
*1 Fraction*				0.9
Mean	23.3	23.1	24.2	
Median	24.2	24.2	24.2	
Range	19.7–25	19.7–25	24.2–24.3	
*2 Fractions*				
Mean	30.8	65	N/A	
Median	30.8	65	N/A	
Range	30.8	65	N/A	
*3 Fractions*				
Mean	N/A	N/A	N/A	
Median	N/A	N/A	N/A	
Range	N/A	N/A	N/A	
*4 Fractions*				
Mean	N/A	N/A	N/A	
Median	N/A	N/A	N/A	
Range	N/A	N/A	N/A	
*5 Fractions*				
Mean	35.7	70	N/A	
Median	35.7	70	N/A	
Range	35.7	70	N/A	
**# Fraction**				
Mean	1.3	1.4	1	
Median	1	1	1	0.6
Range	1–5	1–5	1	
**BED, Gy**				
Mean	118.1	117	126	
Median	126	126	126	0.7
Range	66.7–153.3	66.7–153.3	126	
**SFED**				
Mean	17.2	17.1	18	
Median	18	18	18	0.6
Range	12.7–20	12.7–20	18	
**EQD2**				
Mean	70.8	70.2	75.6	
Median	75.6	75.6	75.6	0.7
Range	40–92	40–92	75.6	
**Isodose Line, %**				
Mean	74.9	74.9	74.5	0.9
Median	75	75	74.5	
Range	65–81	65–81	74–75	
**Follow Up, mo**				
Mean	35.6	36.5	29.5	0.6
Median	39	39	29.5	
Range	6–78	6–78	9–50	

*#, number; BED, biologically effective dose; SFED, single-fraction equivalent dose; EQD2, equivalent total doses in 2-Gy fractions; N/A, not applicable; cc, cubic centimeter; Gy, Gray; mo, months; yrs, years.*

**Table 3:** Radiological and clinical outcomes.

Variables	Entire Series	Pediatric	Adult	Statistical Significance (*p* Value)
LTC				
3 yrs, %	65	67	50	**0.05**
OS				
3 yrs, %	70	75	50	0.24
mean, mo	47.4	47.4	0	
PFS				
3 yrs, %	58	60	50	0.46
mean, mo	42.3	42.3	0	
SI, %	70	75	50	**0.05**
SW, %	10	12.5	50	**0.03**
NS, %	10	0	50	**<0.001**

*LTC, local tumor control; OS, overall survival; PFS, progression-free survival; SI, symptomatic improvement; SW, symptomatic worsening; NS, new symptoms; yrs, years; mo, months.* The bold values indicate statistical significance.

## Data Availability

Data are contained within the article and Supplementary Materials.

## Supplementary Materials

The following supporting information can be downloaded at: https://www.mdpi.com/article/10.3390/jcm13123592/s1, Table S1: Individual Patient Follow-Up Data and Demographic Characteristics.
