# Supplementary material for: The Role of CyberKnife Stereotactic Radiosurgery in Recurrent Cranial Medulloblastomas across Pediatric and Adult Populations
Source: J Clin Med. 2024 Jun 19;13(12):3592. doi: 10.3390/jcm13123592 (PMC11205184; doi:10.3390/jcm13123592)
Supplement: Supplementary file 1 [file jcm-13-03592-s001.zip › jcm-3022620-supplementary.pdf]

Supplementary Table S1. Individual Patient Follow-Up Data and Demographic Characteristics

Pt	Sex	Age	s/p			Tumor		SRS Treatment							Diagnosis to SRS	FU (mo)	Imaging Result	ARE	Death
			SR	RT	CT	Location	Size (cc)	Marginal Dose (Gy)	Fraction	BED	SFED	EQD2	Maximum Dose (Gy)	Isodose Line					
1	M	11	1	1		Cervicomedullary	0.18	18	1	126.00	18.00	75.60	24.16	75	44	29	Stabilized	N/A	
		11				Brainstem	0.02	18	1	126.00	18.00	75.60	24.16	75	44	29	Stabilized	N/A	
2	F	11	1	1	1	Cerebellar	6.97	18	1	126.00	18.00	75.60	24.72	71	29	39	Regression	N/A	
3	M	9	1	1	1	Parietal	1.65	15	1	90.00	15.00	54.00	19.74	76	39	40	Progression	Edema	
		12				Parietal	8.69	20	2	86.67	14.69	52.00	30.77	65	75	78	Stabilized	N/A	
		15				Thalamus	7.2	25	5	66.67	12.72	40.00	35.71	70	108	43	Stabilized	N/A	
		15				Frontal	2.2	16	1	101.33	16.00	60.80	21.05	76	108	43	Regression	N/A	
4	M	13	2	1	1	Frontal	1.13	14	1	79.33	14.00	47.60	20	70	12	20	Progression	N/A	1
		14				Temporal	1.28	20	1	153.33	20.00	92.00	25	80	24	6	Progression	N/A	
5	M	13	1	1		Cerebellar	1.26	18	1	126.00	18.00	75.60	22.5	80	51	17	Progression	N/A	1
6	F	10		1		Cerebellar	2.33	20	1	153.33	20.00	92.00	24.69	81	15	59	Stabilized	N/A	
7	M	29	2			Ventricular	0.47	18	1	126.00	18.00	75.60	24.32	74	24	9	Progression	Necrosis	1
8	M	11	1	1	1	Cerebellar	1.88	20	1	153.33	20.00	92.00	25	80	12	32	Progression	N/A	
9	M	16	1	1		Ventricular	2.71	18.46	1	132.07	18.46	79.24	24.76	75	30	40	Progression	Edema	1
10	M	25	2			Cerebellar	2.5	18	1	126.00	18.00	75.60	24.16	75	25	50	Progression	Necrosis	1

Pt, Patient; M, male; F, female; s/p, status post; SR, surgical resection; RT, radiation therapy; CT, chemotherapy; SRS, stereotactic radiosurgery; BED, biologically effective dose; SFED, single fraction equivalent dose; EQD2, equivalent dose in 2 Gy fractions; FU, follow-up; N/A, not applicable; ARE, adverse radiation effect
